# Assessment of genome annotation using gene function similarity within the gene neighborhood

**DOI:** 10.1186/s12859-017-1761-2

**Published:** 2017-07-19

**Authors:** Se-Ran Jun, Intawat Nookaew, Loren Hauser, Andrey Gorin

**Affiliations:** 1 Department of Biomedical Informatics, College of Medicine, University of Arkansas for Medical Sciences, Little Rock, AR 72205 USA; 2 Comparative Genomics Group, Biosciences Division, Oak Ridge National Laboratory, Oak Ridge, TN 37831 USA; 3 Computer Science and Mathematics Division, Oak Ridge National Laboratory, Oak Ridge, TN 37831 USA

**Keywords:** Genome functional annotation, Gene function similarity, Gene neighborhood, Bayesian probability

## Abstract

**Background:**

Functional annotation of bacterial genomes is an obligatory and crucially important step of information processing from the genome sequences into cellular mechanisms. However, there is a lack of computational methods to evaluate the quality of functional assignments.

**Results:**

We developed a genome-scale model that assigns Bayesian probability to each gene utilizing a known property of functional similarity between neighboring genes in bacteria.

**Conclusions:**

Our model clearly distinguished true annotation from random annotation with Bayesian annotation probability >0.95. Our model will provide a useful guide to quantitatively evaluate functional annotation methods and to detect gene sets with reliable annotations.

**Electronic supplementary material:**

The online version of this article (doi:10.1186/s12859-017-1761-2) contains supplementary material, which is available to authorized users.

## Background

During recent years, technological advances have enabled the rapid and affordable sequencing of organisms from all kingdoms of life. In 2011 the volume of the NCBI Sequence Read Archive crossed a remarkable size of 100 TB [1], and more than 22,000 complete or nearly complete genomes are available for bacterial organisms with the number increasing by >1000 each month [2, 3]. Functional annotation of bacterial genomes is an obligatory and crucially important step of information processing from the genome sequences toward insights into cellular mechanisms, putative ecological roles, or predictive models of a given organism or microbial community. Numerous software packages, databases, platforms, and score filters involve computational pipelines that assign functions to the genes [4]. However, the sequence information is only as good and useful as the functional annotation when it has functional annotation attached to it. The function of genes is central for all biological insights, including interpretation and design of experiments and comparative genomic analysis, as well as the input data for metabolic and regulatory models [5, 6]. The manual curation or experimental verification [7] is unlikely to be feasible when >1000 genomes are added each month. Accordingly, there is a greater urgency to have computational tools for genome annotation validation [8].

In the literature, “annotation quality” sometimes refers to the precision of finding an exact start site for the genes in the genome [8, 9]. When the location of a gene is determined incorrectly, it follows that functional annotation will more likely be incorrect as well. Therefore, the gene finding problem is an important part of the process for genome annotation. In this work, we aim to address annotation consistency at the level where genes are found and annotated by standard protein function annotation, Gene Ontology (GO) terms, organized in a hierarchical fashion [10]. The benefits of function annotation by GO are a systematic control vocabulary that enables cross-comparison over different genomes and a higher percentage of genes in the genome that can be annotated because of different levels of information of GO hierarchy.

In an approach described by Skunca et al. [11], the authors measured the annotation quality of individual GO terms using experimental verifications and estimated the annotation quality of the database UniProt-GOA over time. This approach dealt with relatively small datasets composed of model organisms because it was dependent on experimental verifications. Alternatively, the occurrence of annotation terms was used in a recent computational study [12], which indicated that the manually curated annotations have more natural lexical properties than automatically generated ones, but this method was a bulk analysis within the annotation database and it does not describe the annotation quality of any particular genome. In other studies, authors have used multiple tools and performed manual analysis of the problematic annotations [13, 14]. These are reliable approaches, but they are clearly not scalable to dozens of genomes.

**Fig. 1:**
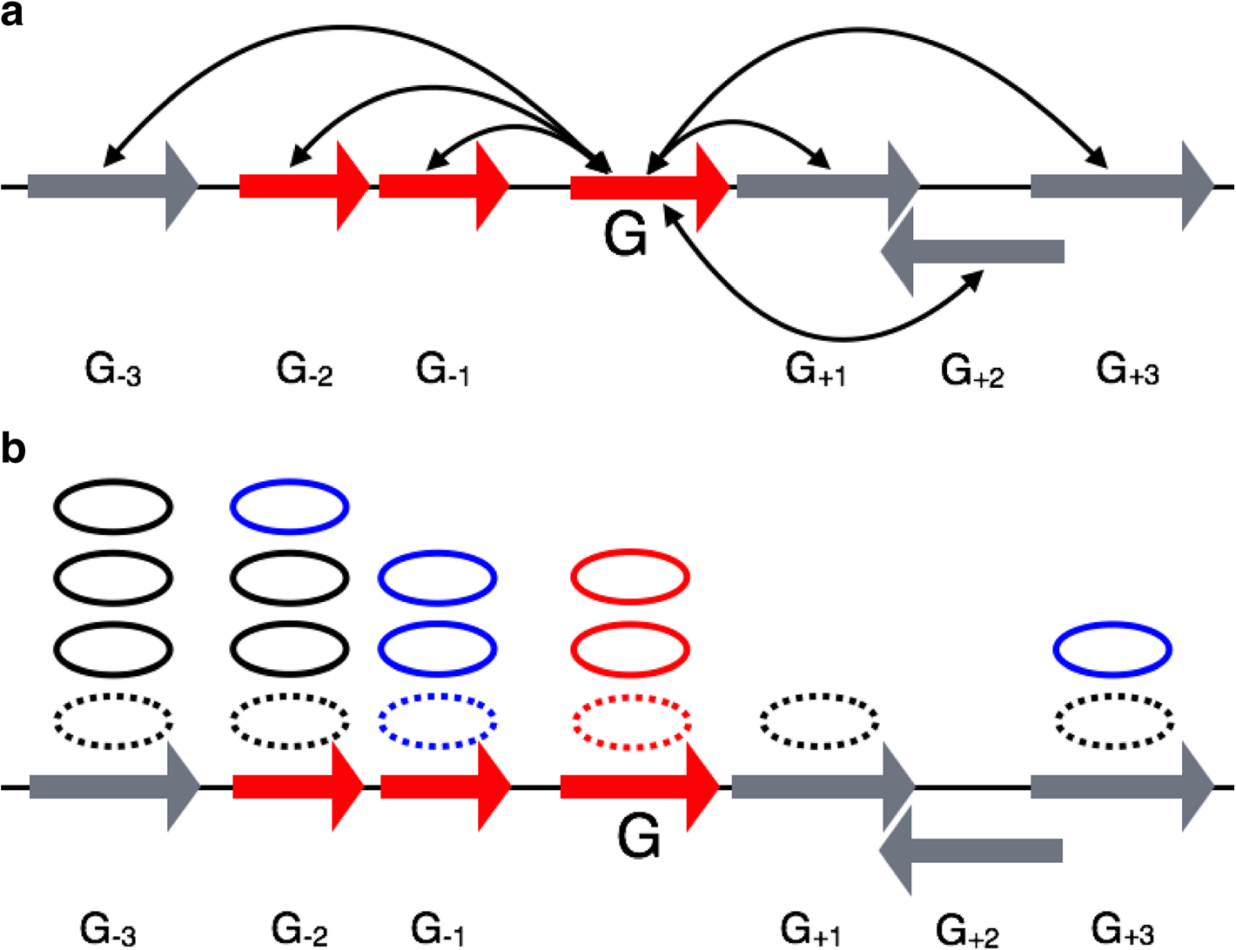
Gene neighborhood and gene function similarity. **a** Gene neighborhood. **b** Gene function similarity. **a** In this study, we looked at three genes in the upstream and downstream directions for neighboring genes of a given gene G. For a gene G, the neighboring gene at +2 is from an opposite strand upstream and genes colored in *red* are organized onto the same operon with the gene G. The functional relationship with neighboring genes within the neighborhood of [−3, 3] is integrated into the formula to calculate PAC where strand and operon information can be integrated into the Eq. (4) (described in Methods). **b** For a pair of two GO terms, function similarity (GOsim) measures how much detailed functional information (low-level GO terms on a GO graph) is shared. All *dotted ovals* represent GO terms assigned to genes where the +2 gene does not have a GO term assigned to it, such that GOsim(G_+2_, G) is not available. All *ovals* over the *dotted ovals* represent predecessor GO terms of assigned GO terms to genes excluding root GO terms on a GO graph. The *ovals* lined in *black* mean that corresponding GO terms do not occur, and the *ovals* lined in *blue* mean that corresponding GO terms occur in a set of predecessor GO terms of a given gene G

Our approach to the validation of gene annotation utilized a well-known and fundamental property of the bacterial genomes: functionally coordinated genes tend to be physically closer on a chromosome than the average gene [15–17]. However, this property was rarely used by others except in a semiquantitative way [18], which used the property to find functional annotations especially for difficult cases of hypothetical proteins. The novel idea of our work (described in Methods in detail) is illustrated in Fig. 1. In this study, a gene neighborhood is defined as three left and right genes of a given gene along the chromosome. We developed an analytical approach to measure gene function similarity (GFS) for each neighboring pair of genes, applied Bayesian statistics to integrate gene neighborhood information of annotation, and then finally, computed the probability of annotation confidence (PAC) for each gene that has at least one GFS score available within its neighborhood, given that functional assignment with very few and well-controlled empirical assumptions is correct. Our method provides genome annotation assessment through the annotation evaluation of all individual genes in the genome.

## Results

### Probability of annotation confidence

**Fig. 2:**
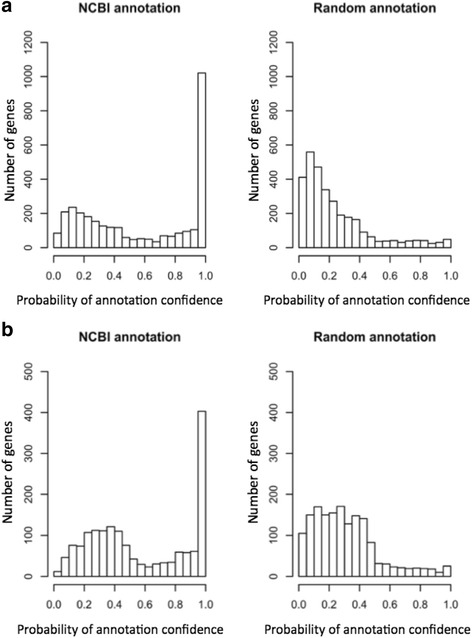
Distributions of PAC values of NCBI and random annotations for (**a**) *E. coli* and (**b**) *C. thermocellum*. **a** Using conditional probabilities derived from a given genome and observed gene function similarities, we calculated PAC values for NCBI annotation (assumed to be correct) and random annotation (assumed to be incorrect) for the *E. coli* strain K-12 substrain MG1655. The probability bin [0.95, 1] has 1021 genes for NCBI annotation and 49 genes for random annotation of 3117 genes applicable to PAC calculation. **b** We applied the same methodology to *C. thermocellum*. The probability bin [0.95, 1] contains 403 genes for NCBI annotation and 25 genes for random annotation among 1617 genes applicable to PAC calculation

**Fig. 3:**
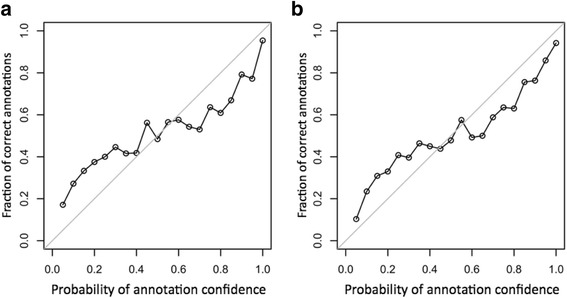
Diagonal plots of fractions of correct annotations for (**a**) *E. coli* and (**b**) *C. thermocellum*. The *x*-axis represents the right-end PAC value for a given bin, and the *y*-axis represents a fraction of correct annotations (NCBI annotations) among all annotations (correct and incorrect) collected for the bin. The points over and under the diagonal indicate overestimation and underestimation of fractions of correct annotations, respectively. In general, we observed points fairly close to the diagonal with both plots

We applied our methodology to *Escherichia coli* and *Clostridium thermocellum* to calculate the PAC for NCBI annotation (assumed to be a well annotation) and compared it with “random” annotation. For each gene with an annotation in *E. coli*, the random annotation was generated by assigning a random annotation selected from 8 million bacterial and archael proteins from UniProtKB/Swiss and UniProtKB/TreEMBL [19] and the NCBI Reference Sequence databases [20]. Note that the random annotation may happen to be correct or partially correct by chance. Figure 2a shows histograms of PAC values (which are Bayesian annotation probabilities described in Methods) for *E. coli* and Fig. 2b for *C. thermocellum* for NCBI annotations and simulated random annotations. For the study in Fig. 2, the simplest model was considered where the independence of function similarities within the gene neighborhood was assumed and information for the operon and strand was not integrated. Note that conditional probabilities derived from each genome were applied to the genome, respectively, for the PAC calculations in Fig. 2. The total number of genes considered in Fig. 2a was 3117 (of 4147 genes), among which 1021 genes had a probability range from 0.95 to 1.00. The distribution of probabilities of the random annotations showed only 49 genes in the probability bin [0.95, 1]. The NCBI annotations with lower PAC values may come from an insufficient number of detectable function similarities with genes in the neighborhood that were derived from the uncovered knowledge of GO annotation and graph structure. We proposed to use a fraction of genes in the probability bin [0.95, 1] as the annotation quality score (AQS) showing distinct differences between NCBI annotation and random annotation. Hence, the NCBI annotation of *E. coli* has an AQS of 0.33 (= 1021/3117) and the random annotation of *E. coli* has an AQS of 0.016 (= 49/3117). The analogous distributions to *C. thermocellum* were plotted in Fig. 2b, and the AQS for *C. thermocellum* NCBI annotation amounted to 0.24, whereas its random annotation had a similar score to *E. coli*, 0.015. We used *C. thermocellum* as an example of a genome that is evolutionarily distant from *E. coli* and most certainly is more difficult to annotate as comprehensively as *E. coli*. The *C. thermocellum* annotation contained a large number of hypothetical genes (~31% of the genome), as well as genes with annotations not fitted into GO classification (~16%). As a result of those adverse factors, only 1617 genes were applicable to a PAC calculation, such that it is reasonable for the AQS for *C. thermocellum* to be lower than the one for *E. coli*, but the difference is not overwhelmingly huge. Figure 3 provides another important assessment for checking the developed methodology. Figure 3a and b accumulated all collected annotations (correct plus incorrect annotation) for each probability bin. The *x*-axis represents the right-end PAC value (Bayesian annotation probability) for a bin and the *y*-axis represents the fraction of true annotations among annotations collected for the bin. On both plots, our model showed a slight overestimation (points over diagonal) and underestimation (points under diagonal) of the sensitivity. However, the probability bin [0.95, 1] showed sensitivity fairly close to the diagonal. Furthermore, both diagonal plots looked almost identical, suggesting the robust properties of the developed methodology even though the annotation of *C. thermocellum* showed sparse functional annotation compared to *E. coli*.

### Operon structure inclusion into the PAC

**Fig. 4:**
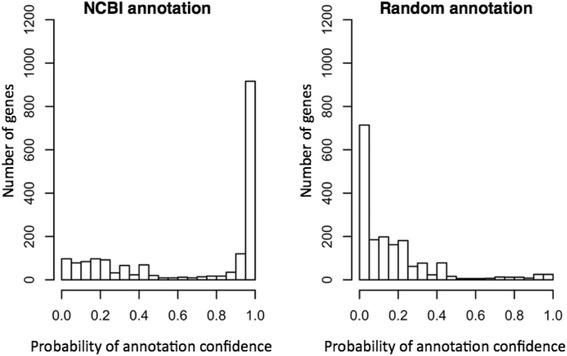
Operon structure inclusion into annotation probability with *E. coli*. The predicted operon information of *E. coli* was integrated in PAC values by considering genes on the same operons for NCBI and random annotation

**Fig. 5:**
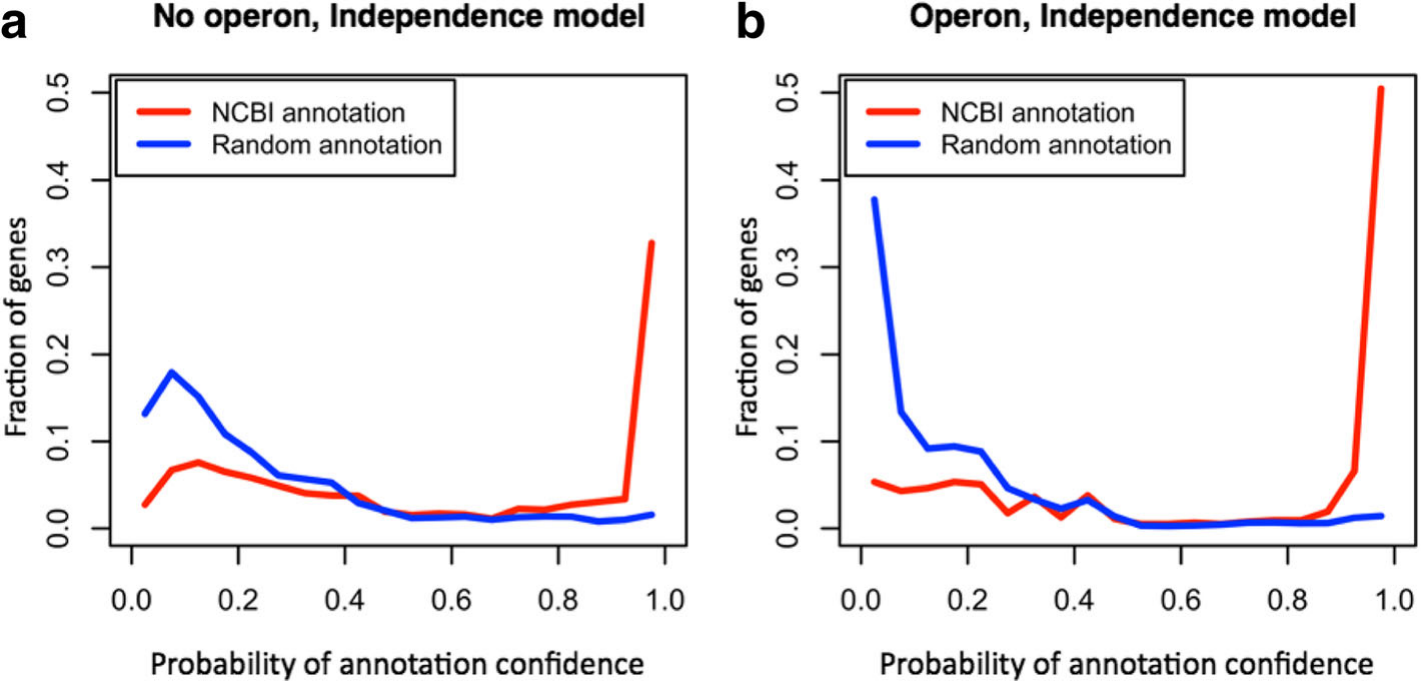
Comparison of no-operon and operon models with *E. coli*. The *y*-axis represents the normalized number of genes within a probability bin by the total number of genes applicable for PAC calculation (**a**) without and (**b**) with operon structure inclusion

So far, we have shown results generated from the simplest model, which used gene function similarities within the gene neighborhood that are assumed to be independent of each other, and clearly distinguished a good quality of annotation from random annotation with the PAC. Yet, a simple integration of the operon structure, which would introduce a separate uncertainty factor in the analysis, could be done by a hybrid system that uses operon-derived conditional probabilities for the genes that are certainly in the same operons and another set of probabilities for the genes that are not. However, in this study, we explored operon structure into PAC by counting only the neighboring genes that are deemed to be on the same operon with a given gene in the formula (4) in Methods. For *E. coli*, inclusion of the operon structure showed rather dramatic changes in the distribution of PAC values in Fig. 4. First, the number of genes with assigned probabilities was reduced significantly because pairs of genes on the same operon were only considered when calculating gene function similarity. The probabilities were assigned only to 1816 genes of 3117 genes in the “no-operon” model. However, there were still 916 genes found in the highly reliable category [0.95, 1] compared to 1021 for the no-operon model (50% of genes for the operon model versus 33% of genes for the no-operon model in the bin [0.95, 1]). The distribution of PAC values in Fig. 4 was much cleaner in a sense that a lower number of genes with PAC values <0.95 were found but still showed a similar shift. However, the distribution for the random annotations had a peak around 0 probability. Summarizing the statement above, Fig. 5 represents the normalized number of genes with PAC values by the total number of genes applicable to PAC calculation for the no-operon and operon models, respectively. Both plots clearly showed that inclusion of the operon structure into our model contributes to a better distinction between NCBI annotation and random annotation.

### Experiments with gene shuffling

**Fig. 6:**
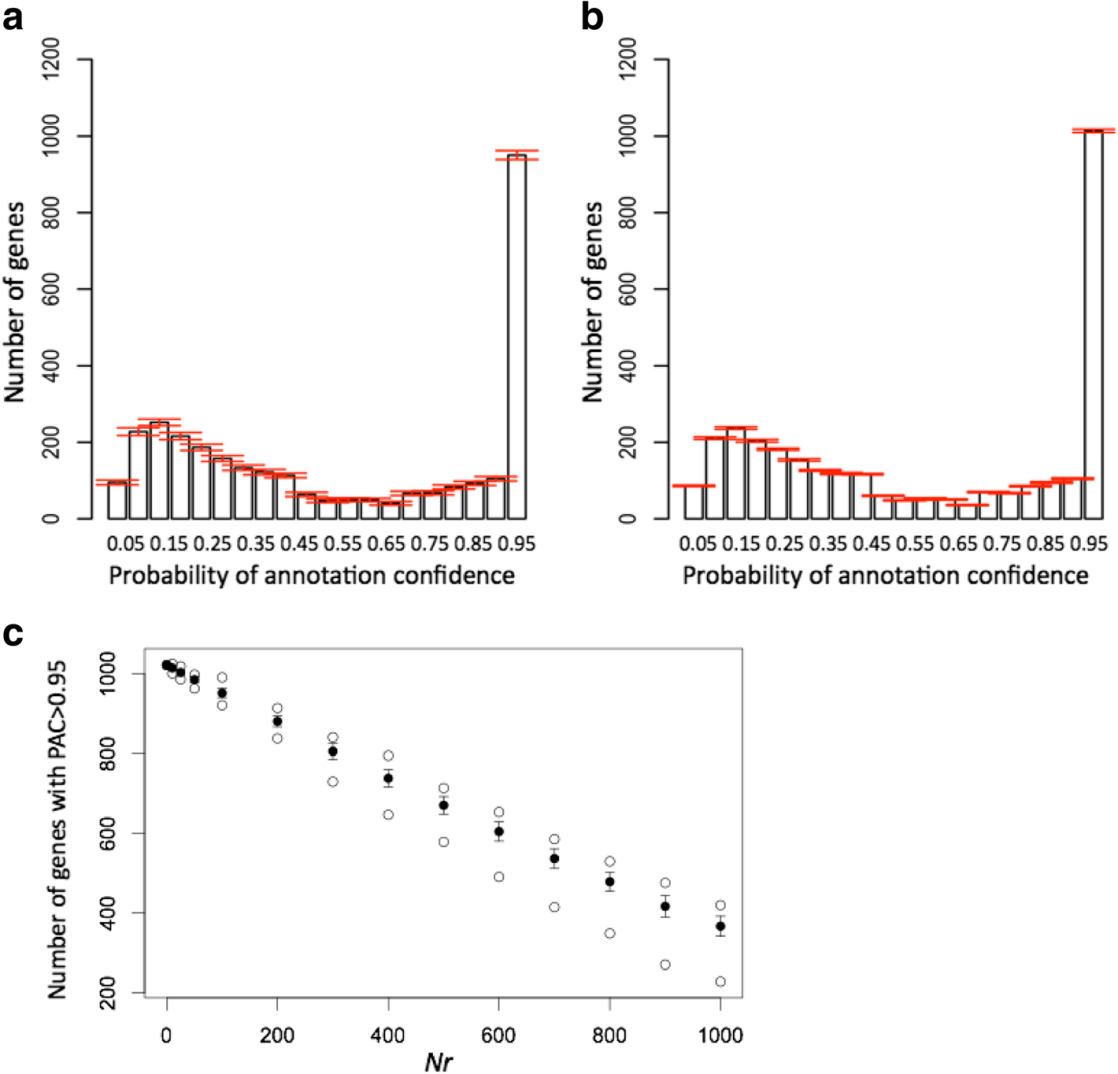
Gene shuffling experiments with *E. coli*. **a** Shuffle for *Nr* = 100. **b** Shuffle for *Nr* = 10. **c** Shuffle summary. **a** and **b** The distributions of PAC values were plotted for the shuffled assignments with *E. coli*. In each experiment, the *Nr* pairs of genes with annotations by GO terms were randomly selected and gene annotations in each pair were exchanged. For each *Nr*, the experiment was repeated 100 times, and the plots represent the average number of genes with the SD observed for each probability bin. **c** The average (*black dot*), SD (*vertical lines*), and maximum and minimum (*white dot*) number of genes were presented for the probability bin [0.95, 1.00] for *Nr* = 10, 25, 50, 100, 200, and up to 1000 (shown on the *x*-axis)

To investigate how our model for annotation validation responds to the increased number of incorrect annotations, we generated annotations with “almost correct functional predictions” through “disturbances by gene shuffling” with NCBI annotation of *E. coli*. In each experiment, we randomly selected *Nr* pairs of genes with annotations by GO terms and exchanged annotations of the selected pairs where annotations were only used once for shuffling. The shuffling procedure was repeated 100 times for each *Nr*. Figure 6 represents distributions of PAC values of the shuffled annotations where each column shows the average number of genes within a probability bin over 100 repeats and the error bars show 1 standard deviation (SD). Figure 6a was constructed for *Nr* = 100, such that 200 genes likely had the wrong annotations. We did not make any additional check on the shuffling process to determine whether it is possible that the shuffling process would swap close or even identical annotations. The SD was small for all probability bins. For example, the average and SD for the probability bin [0.95, 1] were 950.5 and 11.6, respectively, which it is about 6 SD away from the value observed for canonical annotation (1021 genes). In Fig. 6b, we observed that our model remains very sensitive to the annotation disturbance of only 20 genes (*Nr* = 10) for the *E. coli* genome composed of >4000 genes. We had 1013.9 on average with 4.4 SD in the bin [0.95, 1], which is still ~2 SD away from the undisturbed annotation (1021 genes). In Fig. 6c, the average (black dot), SD (vertical line), and maximum and minimum (white dot) number of genes for the probability bin [0.95, 1.00] were presented for *Nr* = 10, 25, 50, 100, 200, and up to 1000 (shown on the *x*-axis). Overall, a linear dependency between the number of shuffling, *Nr*, and a decrease in the (average) number of the genes with highly reliable annotations was observed.

## Discussion

Here we discuss possible enhancements and further developments with potential gains in the model performance: (1) one could explore distance to define neighboring genes as a parameter. For example, one can use basepairs of physical distance along the chromosome as a threshold to define gene neighbors instead of 3 genes upstream and downstream, which is currently used. (2) We treated all genes equally in the current experiments, but in reality the annotations of some genes would be absolutely certain. It would not be difficult to include into our system as another category of genes, “annotation anchors”, and then compute a separate set of conditional probabilities of gene function similarities for such genes. (3) We appended another gene neighborhood structure, “strand information”, into the Bayesian formula with *E. coli* for which we derived conditional probabilities for a set of genes on the same strand and another set of genes not on the same strand. In the Additional file 1: Figure S1 represents PAC distributions calculated from strand-integrated conditional probabilities for NCBI and random annotations, which showed a slightly better performance than those obtained from the model without strand information, in a sense that 1042 genes were found in the bin [0.95, 1] for NCBI annotation, whereas 42 genes for random annotation were found in the bin [0.95, 1]. (4) For all results shown, we extracted the conditional probabilities from Eq. (4) in Methods (likelihood in Bayes’ rule) derived from a given genome. However, *C. thermocellum* was not annotated by functional terms as much as *E. coli* comprehensively, which led to a much lower number of gene pairs with functional annotations, that might not produce enough data to estimate conditional probabilities (likelihood in Bayes’ formula) for probabilistic modeling. To further evaluate robustness toward conditional probabilities, we applied conditional probabilities derived from *E. coli* to calculate the PAC of genes in *C. thermocellum* for NCBI annotation and random annotation. We observed distributions of the PAC values obtained with conditional probabilities derived from *E. coli* similar to those obtained with conditional probabilities derived from *C. thermocellum* in Additional file 1: Figure S2. In the future, we plan to specifically explore this question for a large number of bacterial genomes, yet the result with *C. thermocellum* was very encouraging, even though it is evolutionarily rather distant from *E. coli*. (5) We explored the COG database [21] to annotate genes by functional terms and generated PAC values. Ignoring a poorly characterized functional category, the COG functional terms are organized into three hierarchical levels where the first level consists of three functional classes (Information Storage and Processing, Cellular Processes and Signaling, Metabolism), the finer sub-functional classes (23 functional classes at the second level), and COG terms at the third level. Note that some COG terms belong to more than one functional class. To generate random COG annotation for each protein with an assigned COG term, we assigned a COG term for a protein randomly chosen within the genome to the given protein. The conditional probability of an observation profile given correct and incorrect annotation was calculated for each functional category at the first level where gene COG function similarity takes two values: 0 if two genes share a COG term, and 1 otherwise. In Additional file 1: Figure S3, which represents PAC distributions for NCBI annotation and random annotation with *E. coli*, we obtained an AQS of 0.17 (419/2498 where 2498 proteins were applicable to PAC calculation) for NCBI annotations and an AQS of 0.04 (95/2498) for random annotations that COG annotation showed a less obvious distinction between NCBI and random annotation than GO annotation in the probability bin [0.95, 1]. In the future, we will explore other functional annotation databases including KEGG Orthology [22] and PFAM [23] and compare corresponding PAC distributions for genome annotation validation. (6) So far, we discussed experiments under the “independent” Bayesian model. For example, we approximated the conditional probability of GFSs in the neighborhood as a product of conditional probabilities of individual GFSs within the gene neighborhood. To investigate the influence of the assumption of independence on the AQS, we formulated Bayesian annotation probability under the dependent model, which is described in detail in the Additional files 1 and 2. For the dependent model, we assumed that observations made downstream and upstream depend on only a given gene, and an observation O_i_ depends on an observation O_i+1_ in the downstream and O_i-1_ in the upstream. The distributions of PAC values under the dependent model for *E. coli* are presented in Additional file 1: Figure S4. Under the dependent model considered in this study, we did not observe any gain in terms of the AQS, which is probably due to the assumption not fitting the biological expectation and not enough data to reliably estimate dependency. The main incentive to use it, in any case, is to avoid overestimation and underestimation of PAC calculation, which was not a problem as shown in Fig. 3.

Currently, we envision three possible application directions for the proposed genome-scale model. First, when the different annotation pipelines annotate the same bacterial genomes, our model should be able to compute a measure of consistency for each annotation pipeline; i.e., AQS, the fraction of the genes with a PAC value >0.95. The workflow with a better score would likely have more correct assignments because our genome-scale probabilistic model sensitively captures the small difference in annotations as shown in the Experiments with gene shuffling section. For example, we compared two *C. thermocellum* genomes annotated at different times where one (called old annotation) was annotated on Feb 14, 2007 at GenBank, and the other genome downloaded from NCBI on May 2013 (called new annotation) was used in this study. The old annotation had 1658 proteins (of 3198 total proteins) annotated with GO terms among which 1582 proteins were applicable to PAC calculation, which resulted in 349 proteins in the bin [0.95, 1] leading to an AQS of 0.22 (= 349/1582). The new annotation had 1671 proteins (of 3173 total proteins) annotated with GO terms applicable to PAC calculation, which resulted in 403 proteins in the bin [0.95, 1] leading to an AQS of 0.24 (= 403/1671). The comparison of *C. thermocellum* genomes annotated at different times may support that our model could be a quantitative tool for genome annotation validation. Second, we plan to measure the annotation consistency for many different bacteria (possibly for 32,000 genomes stored by Land et al. [3]), and such research should provide reasonable estimates of which values are reliable for various branches of the tree of life. Finally, individual PAC values should be valuable for the evaluation of hypothetical protein annotation unless functional inference of hypothetical proteins does not exploit gene neighborhood information as happened in other studies [17, 24].

## Conclusions

Sequencing technologies continue to develop rapidly, and the list of genes with assigned functions is the main product of the sequencing efforts, as it is used to further research. However, there is a lack of methods to evaluate the quality of the obtained functional assignments. We developed a genome-scale probabilistic model that quantitatively measures annotation consistency relying on the well-established property of bacterial genomes; i.e., genes lying in physical adjacency on a chromosome tend to be associated functionally. To our knowledge, this is the first tool that provides both a quality value for the whole set of genes as well as probability of the annotation confidence for individual genes in the set. We have tested our method by simulating large and small “disturbances” of the functional assignments, and the method proved to be sensitive for both cases. The range of potential applications is wide including evaluation and comparison of standard annotation methods for functional assignment. This will lead to more biological insights and more precise cellular models as both use functional assignments as input information.

## Methods

### Data

In this study, the genome-scale probabilistic model was first applied to assess the annotation of two genomes: *E. coli* str. K-12 substrain MG1655 (NC_000913.faa) and *C. thermocellum* ATCC 27405 (NC_009012.faa) downloaded from NCBI. The background comparison by random annotation of a genome was performed by randomly picking a protein annotated by functional terms from the protein sequence database. The protein sequence database for random assignments was downloaded from the UniProtKB/Swiss, UniProtKB/TreEMBL [19], and NCBI Reference Sequence [20] databases, which included 8 million bacterial and archeal proteins. The most current version of the same dataset is at least five times as large, but this factor is not important for our particular study.

### GO for functional annotation

To quantitatively assess the annotations, we translated annotations using a controlled vocabulary system, the GO project [10]. The approach to use GO for an evaluation of gene function similarities has been used previously [11, 25], but to our knowledge it has not been used for comprehensive evaluation of genome annotation quality. The GO project describes the ontology of defined GO terms representing gene product properties structured as a directed acyclic graph. The directed graph can be retrieved from “gene_ontology.1_2.obo.txt” [26] which contains GO terms annotated by both the experimental and computational evidence codes. The directed GO graph covers biological process, molecular function, and cellular component, which are mutually exclusive domains each represented by the root GO terms separately. The directed relationships between GO terms represent either “is-a”, “part of”, or “regulates” where child terms are more specialized and parent terms are less specialized. Some GO terms may have more than one parent term unlike a hierarchy. In this work, we considered directed edges, which represent only the “is-a” subclass relationship. The UniProt Gene Ontology Annotation (UniProt-GOA) database provides high-quality GO annotations to proteins through the UniProt Knowledgebase. To annotate NCBI annotations by GO terms, we first assigned NCBI GI numbers to the UniprotKB identifier using “idmapping.dat” [26], and then assigned a UniprotKB identifier into GO terms using “gene_association.goa_uniprot” [27]. Note that the mapping between NCBI GI numbers and UniprotKB identifiers is not one-to-one, and some NCBI GI numbers are not mapped into a UniprotKB identifier.

### Gene function similarity

We introduced GO similarity to compare quantitatively functional annotations described by GO terms. To calculate functional similarity between two GO terms (GO_1_, GO_2_), we first identified a set of all predecessor GO terms of GO_1_ (GO_2_) on the directed GO graph including GO_1_ (GO_2_) but excluding the root, denoted by S_1_ (S_2_), respectively. Then, the similarity between two GO terms was defined based on overlapping GO terms between sets S_1_ and S_2_ as follows:

1 $$ GOsim\left({GO}_1,{GO}_2\right)=\frac{\left|{S}_1\cap {S}_2\right|}{\left|{S}_1\cup {S}_2\right|} $$

where |*S*_1_ ∩ *S*_2_| and |*S*_1_ ∪ *S*_2_| are the cardinalities of an intersection and the union of S_1_ and S_2_, respectively. The normalized GO similarity, which falls in the range of 0 to 1, implicitly measures more than just the detailed functions (low-level GO terms) that are shared. For instance, in Fig. 1b, all dotted ovals represent GO terms assigned to genes where the +2 gene does not have a GO term assigned to it, such that GOsim(G_+2_, G) is not available. All ovals over the dotted ovals represent predecessor GO terms of assigned GO terms to genes excluding the root GO term on a directed GO graph. The ovals lined in black mean that corresponding GO terms do not occur, and the ovals lined in blue mean that corresponding GO terms occur in a set of predecessor GO terms of a gene G. Therefore, GO similarities between neighboring genes and gene G are as follows: GOsim(G_−3_, G) = 0, GOsim(G_−2_, G) = 1/6, GOsim(G_−1_, G) = 1, GOsim(G_+1_, G) = 0, GOsim(G_+3_, G) = 1/4. However, genes can be annotated with more than one GO term because proteins can have multiple functional roles. Let’s say that gene G_1_ is annotated with A_1_ = {GO_i_ |*i* = 1,..,M} and gene G_2_ with A_2_ = {GO_j_ |*j* = 1,..,N}, the GFS between genes G_1_ and G_2_ is defined as the maximum among GO similarities between two GO terms from different genes:

2 $$ GFS\left({G}_1,{G}_2\right)={ \max}_{\begin{array}{c}\hfill 1\le i\le M\hfill \\ {}\hfill 1\le j\le N\hfill \end{array}} GOsim\left({G O}_i,{G O}_j\right) $$

where GO_i_ is from gene G_1_ and GO_j_ is from gene G_2_. The maximum of GO similarities takes into account different numbers of GO terms assigned to different proteins. We calculated the GFS associated with each biological process, molecular function, and cellular component separately.

### Gene neighborhood structure

In this study, we explored three different gene neighborhood structures: gene order on a chromosome, operon structure, and strand information. The strand information of genes was retrieved through the NCBI Entrez Programming Utilities. For the predicted operon structure of *E. coli*, we used the Database of Prokaryotic Operons [28]. For each gene G and each functional category (biological process, molecular function, and cellular component) in a given genome, we calculated GFS(G, G_i_) between G and its neighbor gene G_i_ at *i*th neighborhood, *i* = −3, −2, −1, +1, +2, +3, where the minus and plus signs represent upstream and downstream neighborhoods (Fig. 1a).

### Deriving PAC through Bayes’ rule

Here we derived the probability that annotation of a gene G is correct in given observations {O_i_| *i* = −3,…,+3} with neighbor genes G_i_, *i* = −3,…,+3 (called an observation profile), under the assumption that observations are independent of each other within the gene neighborhood. First, we calculated conditional probability (likelihood in Bayes’ rule) that an observation O_i_ is observed at the *i*th neighborhood given the correct annotation, denoted by Pr(O_i_|A_c_), where A_c_ represents correct annotation, for which NCBI annotation and corresponding functional annotation by GO terms were all assumed to be correct. Then, we calculated the probability that an observation O_i_ is observed at the *i*th neighborhood given the incorrect annotation, denoted by Pr(O_i_|A_inc_), where A_inc_ represents incorrect annotation, for which we generated an annotation for each protein with assigned GO terms by randomly drawing a protein with assigned GO terms from the database of 8 million proteins, and then assigning the GO terms of the randomly drawn protein to the given protein. For each protein, we calculated gene function similarity with gene neighbors using the given gene’s random annotation, leading to Pr(O_i_|A_inc_). If we formulate conditional probabilities using gene function similarity, then a random variable O_i_ takes GFS_i_, where GFS_i_ represents gene function similarity between genes separated by (*i* - 1) genes on a chromosome. The use of combinatorial information of gene neighborhood structures can be easily integrated into the formula. Based on Bayes’ rule along with the assumption of independence of neighbor observations, the probability that an annotation is correct given an observation profile is described as follows:

3 $$ \begin{array}{ll} \Pr \left({A}_c|{O}_i, i=-3,\cdots, +3\right)\hfill & =\frac{ \Pr \left({O}_i, i=-3,\cdots, +3|{A}_c\right) \Pr \left({A}_c\right)}{ \Pr \left({O}_i, i=-3,\cdots, +3|{A}_c\right) \Pr \left({A}_c\right)+ \Pr \left({O}_i, i=-3,\cdots, +3|{A}_{i nc}\right) \Pr \left({A}_{i nc}\right)}\hfill \\ {}\hfill & =\frac{\prod_{i=-3}^{i=+3} \Pr \left({O}_i|{A}_c\right) \Pr \left({A}_c\right)}{\prod_{i=-3}^{i=+3} \Pr \left({O}_i|{A}_c\right) \Pr \left({A}_c\right)+\prod_{i=-3}^{i=+3} \Pr \left({O}_i|{A}_{i nc}\right) \Pr \left({A}_{i nc}\right)},\hfill \end{array} $$

where Pr(A_c_) and Pr(A_inc_) are prior probabilities of correct and incorrect annotations respectively, which were set to 0.5 in this study. By considering all three functional categories concurrently, the Bayesian annotation probability (called the PAC in this study) is described as follows:

4 $$ \begin{array}{ll} \Pr \left({A}_c|{O}_i^{BP},{O}_i^{MF},{O}_i^{CC}, i=-3,\cdots, +3\right)\hfill & =\frac{ \Pr \left({O}_i^{BP},{O}_i^{MF},{O}_i^{CC}, i=-3,\cdots, +3|{A}_c\right) \Pr \left({A}_c\right)}{ \Pr \left({O}_i^{BP},{O}_i^{MF},{O}_i^{CC}, i=-3,\cdots, +3\right)}\hfill \\ {}\hfill & =\frac{\prod_{i=-3}^{i=+3}\prod_{j= BP}^{CC} \Pr \left({O}_i^j|{A}_c\right) \Pr \left({A}_c\right)}{\prod_{i=-3}^{i=+3}\prod_{j= BP}^{CC} \Pr \left({O}_i^j|{A}_c\right) \Pr \left({A}_c\right)+\prod_{i=-3}^{i=+3}\prod_{j= BP}^{CC} \Pr \left({O}_i^j|{A}_{i nc}\right) \Pr \left({A}_{i nc}\right)}\hfill \end{array} $$

where BP indicates biological process; MF, molecular function; and CC, cellular component. For example, if a random variable O_i_ takes a two-dimensional vector of gene function similarity and strand information for each category, then Bayesian annotation probability in the formula (1) is derived from an 18-dimensional observation vector. In most cases, we do not have all neighbor genes with assigned GO terms for all categories. The non-existent information elements are silently ignored in the formula (4) under the assumption that non-existent information occurs equally in correct annotation and incorrect annotation.

### Filtering abundant GO terms

The GFS is affected by GO terms with an abundant occurrence due to their general functional description; for example, GO:0016020, which describes a membrane in a category of the cellular component. Therefore, the GO terms with high frequency can cause random pairs of genes that are not neighbors on a chromosome to share functions, eventually yielding high Bayesian annotation probability. In the Additional file 1; Figure S5 represents the frequency of GO terms in a percentage of proteins with assigned GO terms in the protein sequence database. To avoid false causality with Bayesian annotation probability, we filtered out GO terms whose frequencies were >5%. For 10,000 random protein pairs with assigned GO terms in the protein sequence database, Additional file 1: Figure S6A represents histograms of GFS values before filtering abundant GO terms and Additional file 1: Figure S6B shows GFS values after filtering abundant GO terms with a frequency > 5% in each functional category. In the Additional file 1: Table S1 lists GO terms that were filtered out with a functional description and a 5% of frequency cutoff. All results shown in our study were derived after filtering GO terms with a 5% of frequency cutoff.

## Additional files

Additional file 1: supplementary.doc. Supplementary Figures and Tables. (DOC 947 kb)

Additional file 2: Cthermocellum_oldannotation.txt. The old annotation of *C. thermocellum*. (TXT 364 kb)

## Abbreviations

AQS: Annotation Quality Score
GFS: Gene function similarity
GO: Gene Ontology
PAC: Probability of Annotation Confidence
