# Supplementary material for: Assessment of genome annotation using gene function similarity within the gene neighborhood
Source: BMC Bioinformatics. 2017 Jul 19;18:345. doi: 10.1186/s12859-017-1761-2 (PMC5517811; doi:10.1186/s12859-017-1761-2)
Supplement: Supplementary file 1 — supplementary.doc. Supplementary Figures and Tables. (DOC 947 kb) [file 12859_2017_1761_MOESM1_ESM.doc]

**Assessment of Genome Annotation using Gene Function Similarity within the Gene Neighborhood**

Se-Ran Jun^1^,*

Email: sjun@uams.edu

Intawat Nookaew^1^

[inookaew@uams.edu](mailto:inookaew@uams.edu)

Loren Hauser^2^

Email: hauserlj@ornl.gov

Andrey Gorin^3^

Email: agor@ornl.gov

^1^Department of Biomedical Informatics, University of Arkansas for Medical Sciences, Little Rock, AR, USA

^2^Comparative Genomics Group, Biosciences Division, Oak Ridge National Laboratory, Oak Ridge, TN, USA

^3^Computer Science and Mathematics Division, Oak Ridge National Laboratory, Oak Ridge, TN, 37831, USA

*Corresponding author

**Figure S1.** Strand-integrated PAC distributions with *E. coli*. PAC values were calculated from strand-integrated conditional probabilities, which were derived for a set of genes on the same strand and another set of genes not on the same strand, respectively. The strand-integrated PAC distributions showed a slightly better performance than those obtained from the model without strand information, in a sense that 1042 genes were found in the bin [0.95, 1] for NCBI annotation, whereas 42 genes for random annotation were found in the bin [0.95, 1].

**
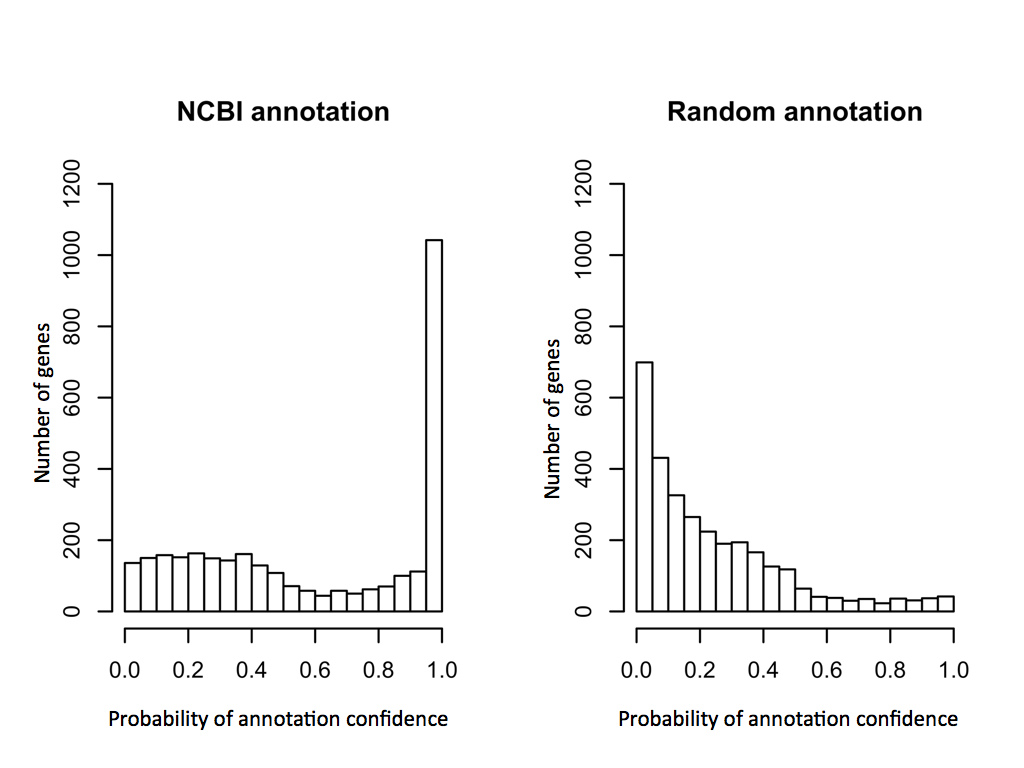
**

**Figure S2.** Distributions of PAC values for *C. thermocellum* computed with the conditional probabilities (likelihood in Bayes’ rule) derived from *E. coli*. The distributions were similar to those obtained from conditional probabilities derived from *C. thermocellum*.

**
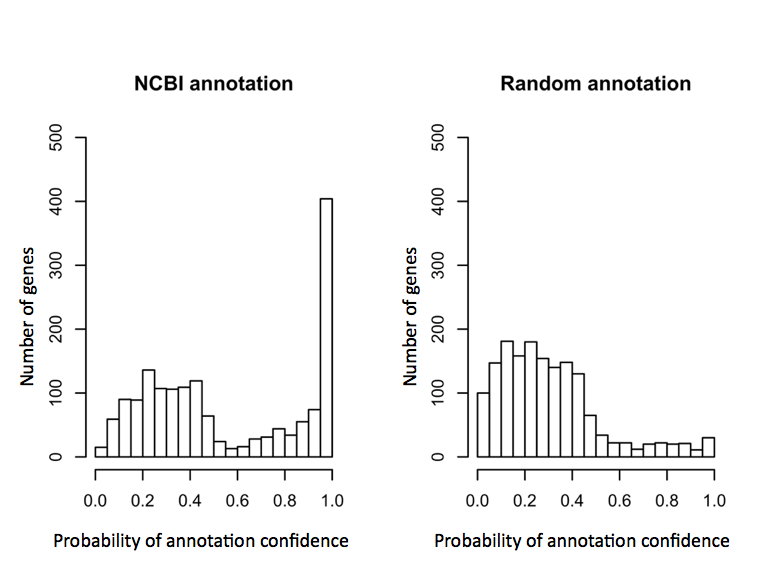
**

**Figure S3.** Distributions of PAC values by COG annotation with *E. coli*.


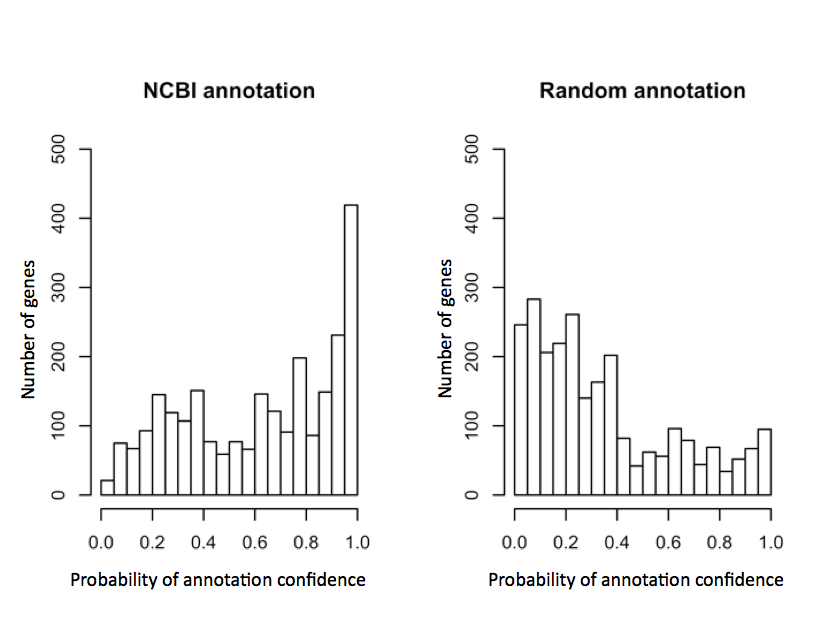


**Dependent model**

For the dependent model, we first assumed that for a given gene G, an observation profile, X = {O_i_|*i* = -3,-2,-1}, in the left-neighborhood depends on only the gene G, and an observation profile, Z = {O_i_|*i* = +1,+2,+3}, in the right-neighborhood depends on only the gene G. Therefore, the probability of an observation profile given an annotation (*A*) of the gene G is described as follows:

. (1)

Second, we assumed that O_i_ depends only on O_i+1_ in the left-neighborhood and only on O_i-1_ in the right-neighborhood, depicting that annotation information of the gene G influences into genes in order, in both directions (left- and right-neighborhood):

$\begin{matrix} \Pr(O_{-3},O_{-2},O_{-1}|A)=\Pr(O_{-1}|A)\Pr(O_{-2}|A,O_{-1})\Pr(O_{-3}|A,O_{-2}) \\ \Pr(O_{+1},O_{+2},O_{+3}|A)=\Pr(O_{+1}|A)\Pr(O_{+2}|A,O_{+1})\Pr(O_{+3}|A,O_{+2}) \end{matrix}$. (2)

Therefore, the Bayesian PAC given for an observation profile is derived by plugging equation (2) into the following equation:

. (3)

Under the dependent model, to derive the conditional probability (likelihood in Bayes’ rule), for example$\Pr(O_{i}|A,O_{i+1})$, we need to examine 100 different cases if we want to bin the gene function similarities into 10 different groups. Due to the data limitation, we binned gene function similarity into 2 different groups as follows:

$O_{i}=\left\{\begin{matrix} 0 & if\text{ }O_{i}\leq0.5 \\ 1 & otherwise \end{matrix}\right.$.

Figure S4 shows the distribution of PAC values for *E. coli* under the dependent model described above. In this study, we did not observe any gains from using the dependent model considered, which is probably due to the imposed restriction not fitting biological expectations and not enough data to capture the dependency. However, the main incentive to the dependent model, in any case, is to avoid overestimation and underestimation of PAC calculations, which was not a problem (as shown in Figure 3).

**Figure S4.** Distributions of PAC values derived from the dependent model with *E. coli*.


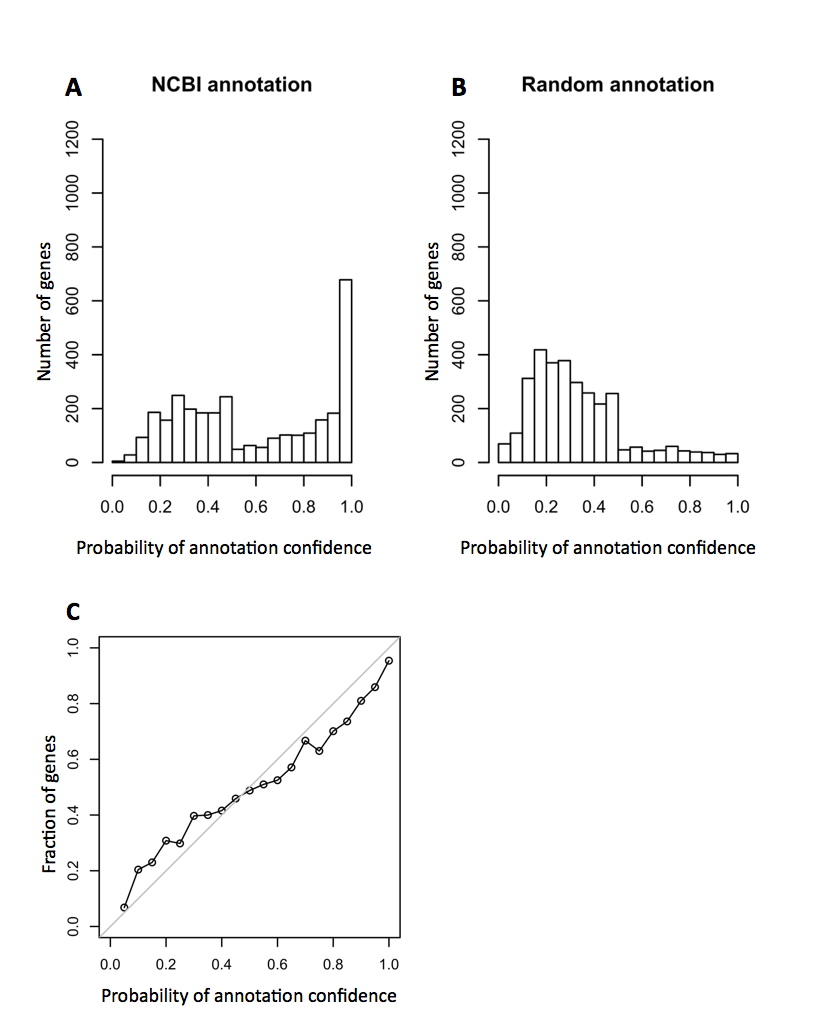


**Filtering abundant GO terms**

**Figure S5.** GO frequency. The *y*-axis represents the percentage of proteins with GO terms assigned to them in the protein sequence database for each category.

**Figure S6.** Distribution of GFS values for 1000 random protein pairs. (A) Before filtering abundant GO terms and (B) after filtering abundant GO terms with a 5% frequency cutoff.

**
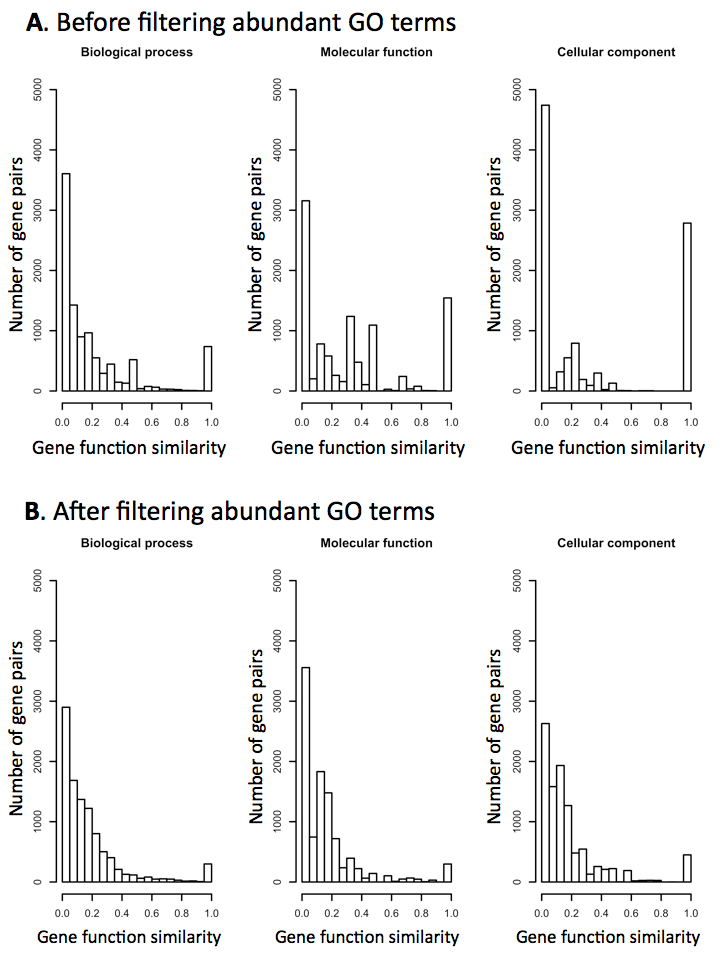
**

**Table S1.** List of GO terms filtered by 5% frequency cutoff.

| Biological Process | Name |
| --- | --- |
| GO:0055114 | oxidation-reduction process |
| GO:0008152 | metabolic process |
| GO:0006355 | regulation of transcription, DNA-dependent |
| GO:0006351 | transcription, DNA-dependent |
| GO:0006810 | transport |
| Molecular Function | Name |
| GO:0016740 | transferase activity |
| GO:0003824 | catalytic activity |
| GO:0016491 | oxidoreductase activity |
| GO:0016787 | hydrolase activity |
| GO:0005524 | ATP binding |
| GO:0000166 | nucleotide binding |
| GO:0003677 | DNA binding |
| GO:0003700 | sequence-specific DNA binding transcription factor activity |
| GO:0046872 | metal ion binding |
| GO:0005215 | transporter activity |
| Cellular Component | GO Name |
| GO:0005737 | cytoplasm |
| GO:0005840 | ribosome |
| GO:0005886 | plasma membrane |
| GO:0016020 | membrane |
| GO:0005622 | intracellular |
| GO:0030529 | ribonucleoprotein complex |
| GO:0016021 | integral to membrane |
